# Self-optimisation and model-based design of experiments for developing a C–H activation flow process

**DOI:** 10.3762/bjoc.13.18

**Published:** 2017-01-24

**Authors:** Alexander Echtermeyer, Yehia Amar, Jacek Zakrzewski, Alexei Lapkin

**Affiliations:** 1 Aachener Verfahrenstechnik – Process Systems Engineering, RWTH Aachen University, Aachen, Germany; 2 Department of Chemical Engineering and Biotechnology, University of Cambridge, Cambridge, United Kingdom

**Keywords:** automated reaction system, C–H activation, design of experiments, flow chemistry, process modelling, self-optimisation

## Abstract

A recently described C(sp^3^)–H activation reaction to synthesise aziridines was used as a model reaction to demonstrate the methodology of developing a process model using model-based design of experiments (MBDoE) and self-optimisation approaches in flow. The two approaches are compared in terms of experimental efficiency. The self-optimisation approach required the least number of experiments to reach the specified objectives of cost and product yield, whereas the MBDoE approach enabled a rapid generation of a process model.

## Introduction

**Figure 1:**
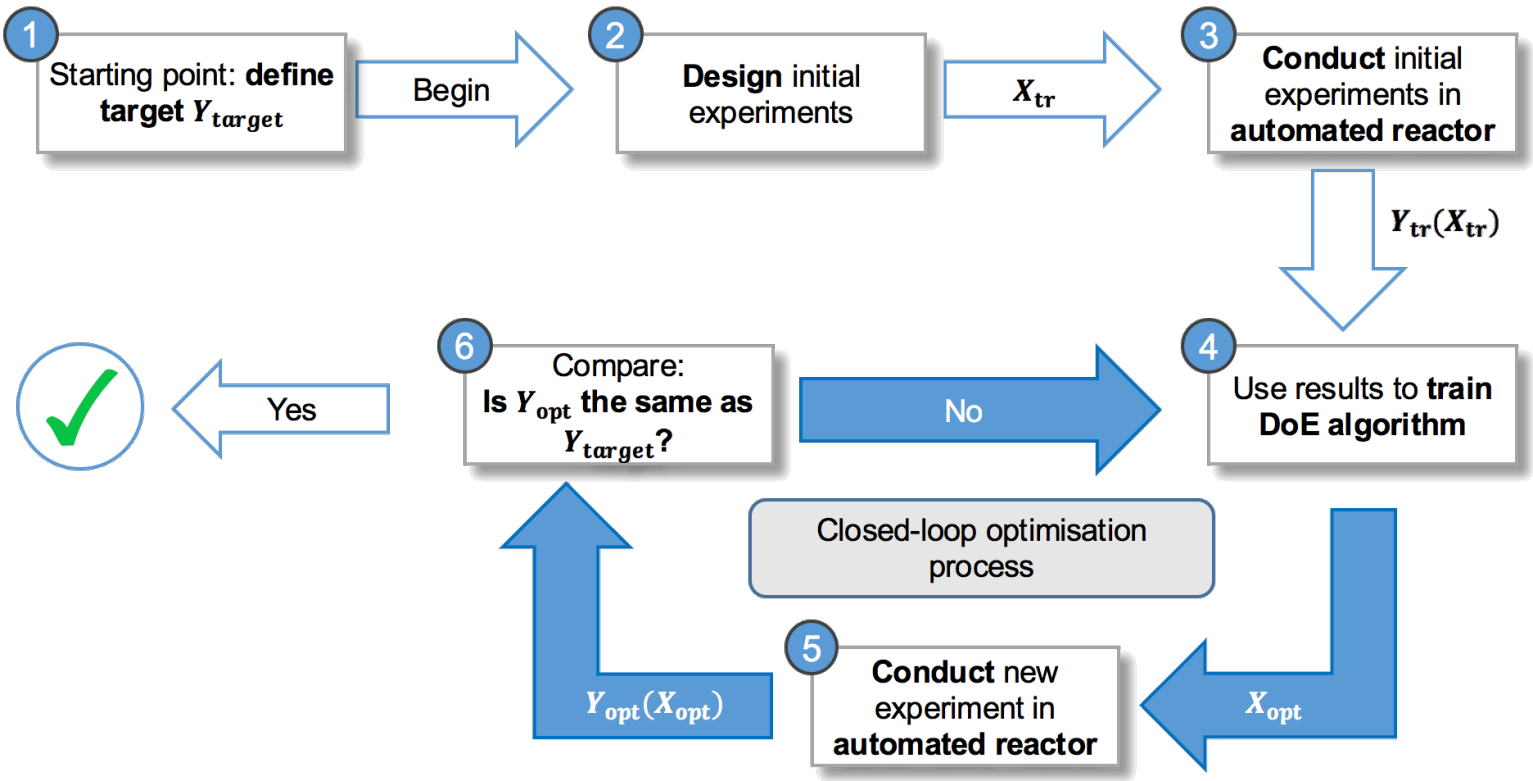
A framework of closed-loop or self-optimisation combining smart DoE algorithms, process analytics, chemoinformatics and automated reactor systems.

The development of manufacturing processes to produce functional molecules, such as pharmaceuticals or fine chemicals, often relies on experience and trial-and-error, rather than on mechanistic process models [1]. The only reason for this is the complexity of chemistry and the duration of time required for the development of good mechanistic models. A game changer in this area is the recently emerged field of automated continuous-flow experiments driven by algorithms for sequential design of experiments (DoE), which significantly reduce the effort in running routine reactions and generating data for optimisation of reaction conditions [2–7]. An illustration of the concept is shown in Figure 1.

Mainly, self-optimisation experimental platforms are used to rapidly obtain optimal reaction conditions using either flow [8–10] or batch experiments [11]. In these cases, the optimisation is driven by the global or target optimisation towards the selected performance criteria. This is rather different from the objectives of model development. In the case of model development, the key criterions are the ability of a model to describe the observed experimental data and to predict process performance under unseen conditions. Thus, experiments required for model development are frequently what would be considered as ‘bad’ experiments in the case of optimisation.

A model-development framework has been demonstrated on the basis of an automated microreactor experimental system for several complex reactions [8,12–13]. The framework uses factorial design of experiments to obtain an initial data set for parameter estimation, followed by an iterative search with online model discrimination and parameter estimation, guided by D-optimal design. In a different approach, transient data from continuous-flow experiments were used to identify parameters of a known mechanistic scheme to discriminate between several alternative model structures and to identify model parameters, but no specific design of experiments method was used [14].

The framework proposed in the present publication is using a model-based design of experiments method (MBDoE) [15–17], which incorporates the model with its parameters, as well as details of the experimental setup, such as measurement accuracy and experimental limitations, to design the most informative experiments. This approach requires some model structures to be known a priori which may restrict the methodology to reactions with known mechanism, or to empirical parametric models. A discussion of how a priori knowledge of chemistry, i.e., reaction mechanisms, is included in self-optimisation and model-development frameworks is not well documented in the literature. Very recently we have shown that a priori knowledge in the form of density functional theory level (DFT) mechanistic calculations can be used to propose process models and to perform in silico design of novel flow processes [18]. In this publication, we present an extension of this methodology, in which an initial process model is developed through a MBDoE methodology coupled with an automated self-optimisation flow system.

**Scheme 1:**
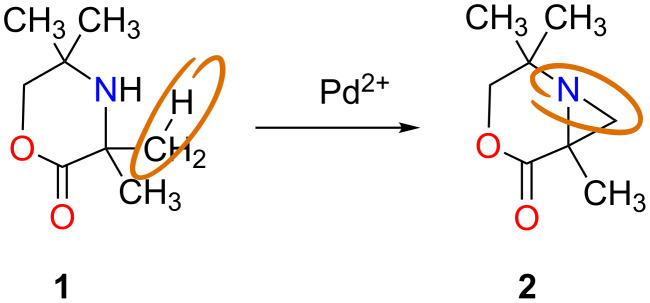
Catalytic reaction scheme showing C–H activation of an aliphatic secondary amine **1** to form the aziridine product **2** [19–20]. Orange rings show C–H and C–N bonds in the substrate and the product, respectively, indicating the location of the C–H activation.

**Scheme 2:**
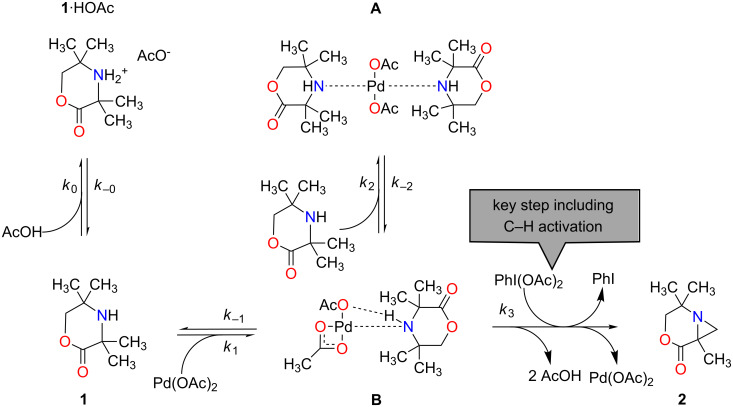
A simplified reaction mechanism based on literature [21], showing intermediate **B** and the side reaction compounds **1**∙HOAc and **A**. The key step includes the C–H activation. **1**: starting material, **1**∙HOAc: coordinated starting material, Pd(OAc)_2_: catalyst, **2**: product, PhI(OAc)_2_: oxidant.

This approach was tested on the Pd-catalysed C–H activation reaction of **1** resulting in the formation of an aziridine **2** (Scheme 1) [19]. The reaction was recently discovered [20] and its mechanism studied [21] and later proven [18]. A simplified mechanism is shown in Scheme 2. In the reaction of interest, the starting material **1**, an aliphatic secondary amine, is converted into an intermediate species **B** in a catalytic first step and consecutively transformed to product **2** in the second step, which comprises the C–H activation. In addition to the main reaction pathway, **B** can form the relatively unreactive resting state complex **A**, and compound **1** can also form a coordinated species **1**∙HOAc upon protonation with a molecule of acetic acid. This limits the formation of **A** due to reduced concentration of **1**.

Table 1 gives an overview of the a priori knowledge used in this study. Fast reaction steps were lumped into a single one, containing the critical C–H activation, and described by reaction rate constant *k*_3_ in Scheme 2. Empirical information provided constraints of process conditions, such as temperature and concentration ranges, whereas initial values of kinetic parameters were estimated based on a DFT model. Further details can be found in Supporting Information File 1.

**Table 1:** Details of information considered as a priori knowledge in this study and source of this knowledge.

A priori knowledge	Source

reaction mechanism, concentration constraints of species due to degradation of starting material and product.	[21]
Gibbs free energies of reaction, obtained from DFT study.	[18]
target values based on best results from previous experimental study.	[18]
physical constraints (maximum oxidant concentration to prevent crystallisation, maximum temperature to prevent excessive catalyst decomposition).	empirical
technical details of experimental set-up (e.g., variance of gas chromatography (GC) used in variance model for MBDoE, minimum and maximum flow rates).	empirical

Here we demonstrate an MBDoE approach on the basis of the model structure and the initial model parameters from DFT calculations and using automated flow experiments. We then use the obtained process model to develop a surrogate model for optimisation, and compare the different methodologies: classical kinetic modeling approach, MBDoE with automated flow experiments and black-box optimisation in achieving the different objectives of the methods.

## Results and Discussion

### Experimental system for model development and optimisation in flow

**Figure 2:**
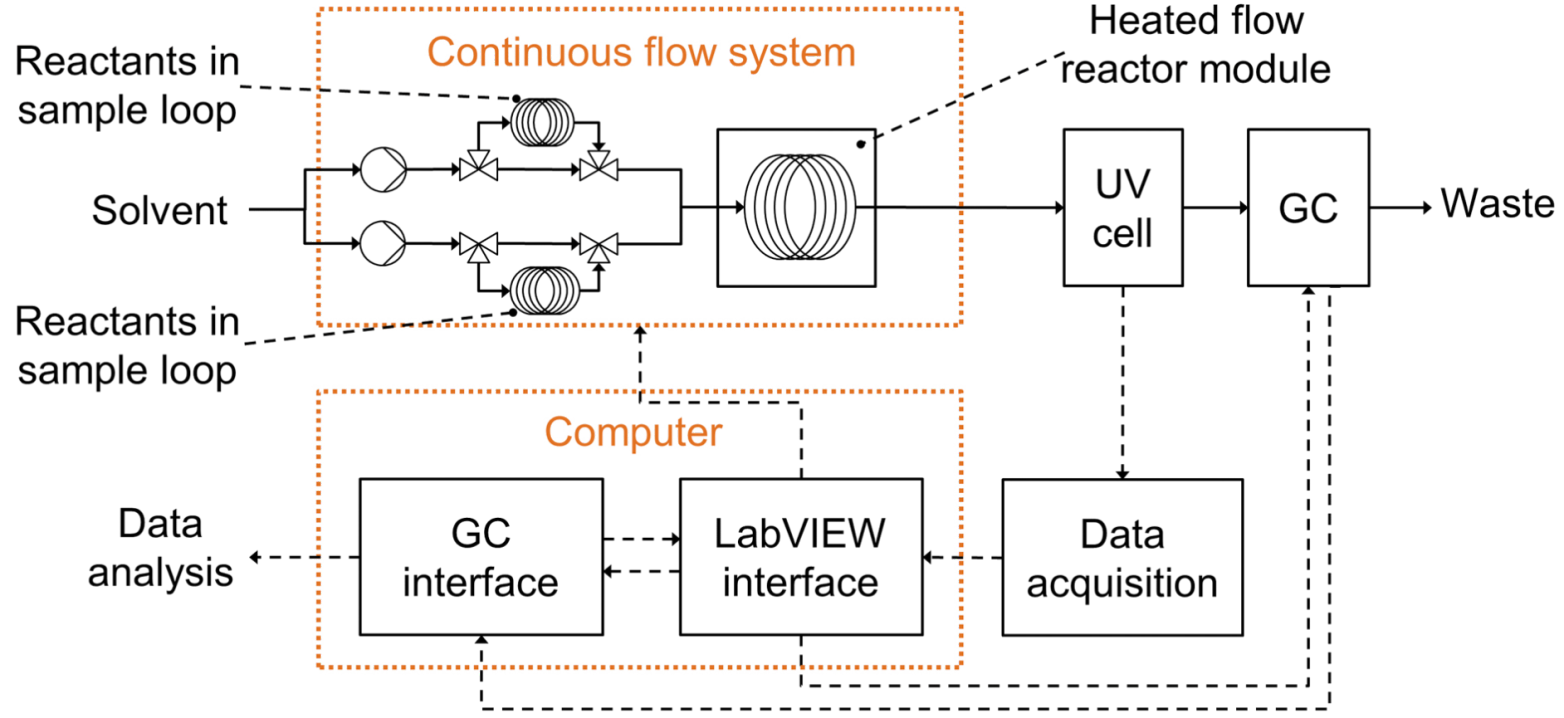
Schematics of the automated continuous-flow system used for model development and ‘black-box’ sequential optimisation.

Although a number of experimental systems for self-optimisation were reported in the literature, this number is fairly small and very few examples of using flow experiments for model development are reported [8–9]. In this study a commercial Vapourtec R2+/R4 system was used with a standard 10 mL coiled reactor. To save on expensive reagents, reagents and catalyst were injected using 2 mL sample loops, with the solvent being continuously pumped between the reaction slugs. The two employed sample loops were filled with the same reaction mixture (further information on sample preparation is given in Supporting Information File 1) to avoid potential experimental errors due to inaccuracies of generating mixtures with specific concentrations by pumps. Laminar flow through long pipes will necessarily cause dispersion, which dictates the minimum reaction slug length that can be used. This was determined experimentally, which also allowed to develop the method of detection of the reaction slugs (by a flow UV cell) and the protocol for GC sampling. A schematic depiction of the experimental system is shown in Figure 2.

### Physical model generation and refinement

The initial model structure and parameters were taken from the earlier published DFT study of the reaction [18]. Performing MBDoE in the process modelling software gPROMS [22] resulted in a design indicating the experimental conditions, the reaction times and the number of samples required in each experiment for the estimation of a particular parameter or the combination of parameters. Table 2 shows the different experiments conducted for estimation of the given parameters. Each experiment refers to a particular composition of the reaction mixture, but with various reaction times for each sample within the experiment. Neither in the MBDoE step for *k*_j,ref_ nor in the step for *E*_a,j_ could an experiment be designed for the estimation of all parameters simultaneously. This is likely due to correlations between the parameters, which is common for reaction networks and consecutive reactions. To overcome this problem, sophisticated decoupling techniques and special design criteria considering direct measures of correlation could be used [23–24]. However, as shown by Franceschini and Macchietto, a simple design-by-grouping method can also yield reasonable results [25]. Following this approach experiments were designed for either a single or groups of parameters. Parameters, which showed a maximum in their normalised local sensitivity curves in the same time interval were grouped together. This is reasonable, as a sample taken in this time interval likely yields sensible data for the estimation of the respective parameters. As can be seen from Figure S9 (Supporting Information File 1), all parameters of the same type showed maximum sensitivity in approximately the same time interval. Hence, all possible combinations of single and grouped parameters were tested in the two MBDoE steps and those with the lowest correlation, maximum number of included parameters and a t-value larger than a reference t-value were selected. It is worth noting, that this method overcomes problems with parameter correlations during the experimental design for parameter subgroups and the subsequent estimation. The effects of the neglected parameter correlation may reoccur during the overall parameter estimation, but can be reduced due to the refinement of the parameters in the subgroups.

**Table 2:** Results of the MBDoE for kinetic parameters, showing the number of samples needed in the experiment and the statistical t-test results.

Experiments	Parameter(s)	Number of samples	t-value	*t*_ref_

1	*k*_0,ref_	7	76.19	2.92

2	*k*_2,ref_	6	23.36	2.92
3	*k*_3,ref_	5	23.36	2.92
4	*k*_0,ref_, *k*_2,ref_, *k*_3,ref_	11	5.34, 0.03, 6.42	1.94
5	*E*_a,0_, *E*_a,2_, *E*_a,3_	11	0.05, 0.04, 2.88	1.94
6	*E*_a,0_, *E*_a,2_, *E*_a,3_	11	0.63, 0.25, 2.33	1.94
7	*E*_a,0_, *E*_a,2_	10	2.79, 17.1	2.02
8	*E*_a,0_, *E*_a,3_	10	3.99, 46.8	1.94

The best possible design with minimum analytical effort was selected. It can be seen from Table 2 that the t-test is successful for the experiments 1–3. This is not surprising as possible correlations between the parameters are neglected by splitting them into subgroups or even singles. For the experiments 4, 5 and 6 (Table 2) not all parameters pass the t-test. The best possible experimental design was selected. Due to failed estimability analysis, no experiment design included parameters for the reaction *j* = 1. Experimental conditions associated with each experiment sequence are given in Supporting Information File 1, Table S4 and Table S5.

### Parameter estimation and comparison of effort

For the investigated reaction in Scheme 2, Zakrzewski et al. generated and validated a kinetic model using a classical kinetic approach [18]. For this they used 38 batch experiments, each comprising approximately 10 sample points at different reaction times, which in total resulted in more than 400 sample points used for the estimation of kinetic model parameters. In contrast to that, we used MBDoE and flow experiments. As Table 2 shows, the MBDoE resulted in 8 experiments with a total of 71 samples required to determine the model parameters. These numbers highlight the benefit of MBDoE for parameter estimation, reducing the consumption of materials, cost and time associated with sample generation. Due to some failed experiments only 64 experimental sample points were used for the model development.

The parameter estimation was employed to obtain estimates of the kinetic parameters *k*_j,ref_ and *E*_a,j_*,* where *j* 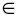 {0, 1, 2, 3} in a two-step procedure using standard solver settings in gPROMS. By applying the initial guesses for the parameters, each experiment was first used to estimate only the parameter for which it was designed, while keeping the others fixed at their current values. Afterwards, all experiments were included in an overall estimation with the parameter values obtained from the previous estimations as new initial guesses to account for possible parameter correlations, which were neglected by grouping the parameters. Even though no experiment design comprised the parameters for reaction *j =* 1 specifically, they were still included in the overall estimation to refine their initial values as much as possible. To avoid stopping the estimation at undesired local optima, several such estimation runs were performed. The final results of the obtained parameter values are shown in Table 3. The final values of parameters *k*_1,ref_ and *E*_a,1_ do not differ much from the initial guesses, which is not surprising as the estimability analysis had already predicted a weak influence of *k*_1,ref_ and *E*_a,1_ on the model output, i.e., this cannot be estimated with precision. However, this was not necessary, as the parameters do not change the model prediction. Therefore, also the very large 95% confidence interval can be explained. For all other parameters, the difference between initial guess and final value is significant, which might be caused be the simplifications employed for computing the guesses and the uncertainty of the DFT calculation in the exponentially amplified van’t Hoff equation. The 95% confidence intervals for the parameters of reaction *j =* 0 are both one magnitude smaller than the final parameter values indicating sufficiently low uncertainty and good significance. The confidence intervals for the parameters of reaction *j* 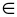
{2, 3} are larger than the final parameter value. However, as the values for *k*_j,ref_ and *E*_a,j_ can only be positive, this indicates still some level of uncertainty in the parameters estimates. This uncertainty is further revealed by a comparison of the t-values and the reference t-values. For the overall estimation of *k*_j,ref_ it was impossible to attain t-values exceeding the reference t-value, even though *k*_0,ref_ comes close. Furthermore, the predicted t-values from the MBDoE, shown in Table 2, could not be reached. In the overall estimation of *E*_a,j_ three out of four parameters could not be estimated with high statistical significance. Only for *E*_a,0_ the t-test was satisfied with a t-value close to the predicted one in Table 2. The problem of diminished statistical significance of the estimates is likely due to practical identifiability issues as the measurement data employed for the estimation was affected by experimental errors. Additionally, parameter correlation effects reappeared during the overall estimation making it more difficult to obtain useful results.

**Table 3:** Results of the parameter estimation showing the final values, initial guesses, 95% confidence intervals (CI) and t-values for each of the 8 parameters.

Parameter	Initial guess	Final value	Units	95% CI	t-values

*k*_0,ref_	3.019	3.035	L mol^−1^ s^−1^	±0.396	1.403^a^
*k*_1,ref_	2,551,604	2,728,600	L mol^−1^ s^−1^	±4.817∙10^11^	2.49∙10^−6 a^
*k*_2,ref_	8,591	16,997	L mol^−1^ s^−1^	±6.282∙10^5^	0.012^a^
*k*_3,ref_	0.001756	0.140378	s^−1^	±8.175	0.012^a^
*E*_a,0_	84,132	128,517	J mol^−1^	±5.152∙10^4^	2.495^b^
*E*_a,1_	45,019	44,941	J mol^−1^	±1.709∙10^10^	2.63∙10^−6 b^
*E*_a,2_	59,508	20,995	J mol^−1^	±3.525∙10^6^	0.006^b^
*E*_a,3_	98,831	144,942	J mol^−1^	±3.517∙10^6^	0.041^b^

^a^Refers to *t*_ref_ = 1.725 and ^b^refers to *t*_ref_ =1.688.

**Figure 3:**
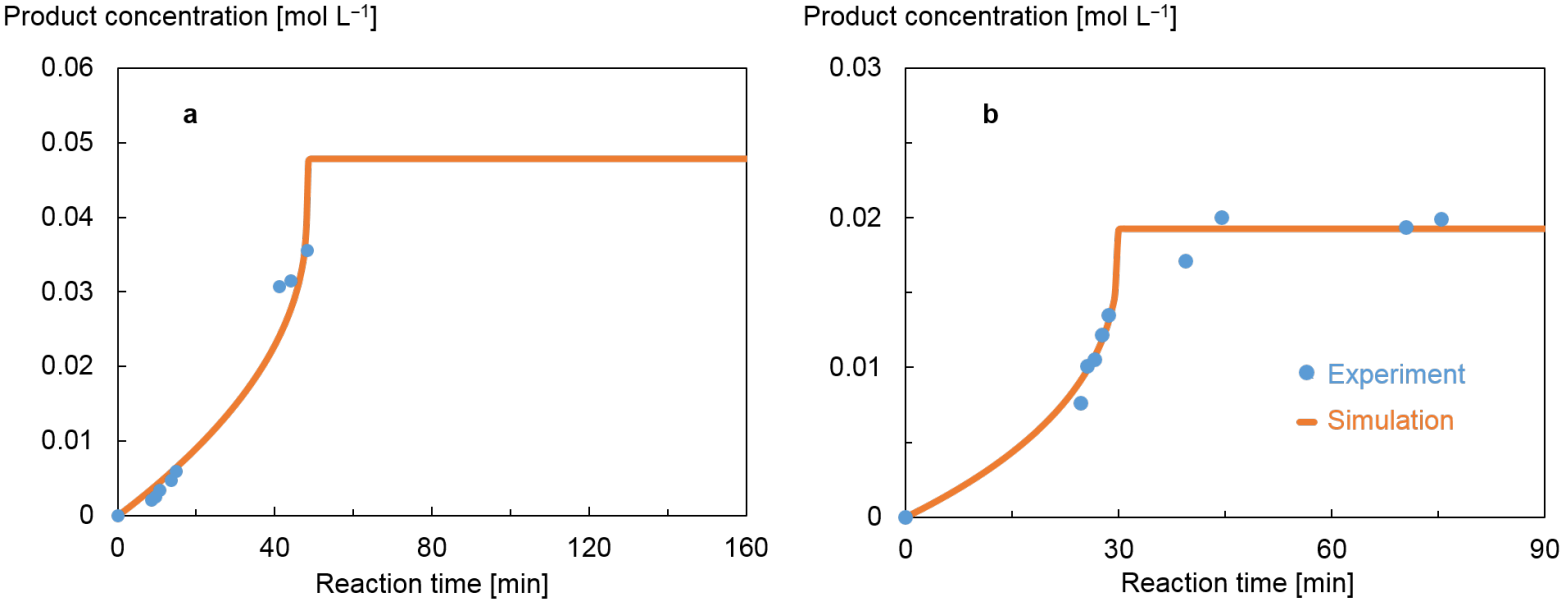
Results of experiments from the MBDoE in Table 2, conducted for parameter estimation, and their corresponding simulated model responses based on the estimated parameters. The only experiments conducted were those calculated by the MBDoE, defined by the sampling times and recipes suggested. (a) Experiment 4 conducted at a reference temperature of 70 ºC to estimate rate constants. (b) Experiment 8 used for determining activation energies, at 75 ºC.

Figure 3 shows a comparison of the simulated model response incorporating the final parameter values vs the experimentally observed product concentrations for the experiments 4 and 8 of the MBDoE in Table 2. These show a reasonably good model fit. Only experiments suggested by MBDoE were conducted to generate data for parameter estimation. Thus, as the method did not suggest samples to be taken at reaction times longer than 50 minutes in experiment 4, Figure 3a, or between zero and 24 minutes in experiment 8, Figure 3b, there was no data collected. In total, 8 such experiments were conducted, four for each of the two parameter types (shown in Supporting Information File 1).

Despite the remaining uncertainty in some of the parameters, indicated by the large 95% confidence intervals, the quality of model prediction was considered to be good-enough for the purpose of in silico training of the smart DoE algorithm for target optimisation. Thus, the final parameter values in Table 3 were accepted and used in the model employed for the subsequent in silico target optimisation steps.

### Improvement of process conditions using an a priori model and in silico optimisation

Access to automated experimental systems allows to perform black-box sequential optimisation using sequential DoE algorithms. However, if a process model is available, there exist two more options for optimisation: optimisation using the available process model directly, or optimisation using a surrogate model. The latter is frequently used in expensive computer experiments, and in the case of large-scale process simulations, when evaluations of process models is computationally too expensive. In the case of our test reaction the MBDoE approach enabled us to develop a reasonably good process model in a small number of flow experiments. We can use this process model to perform optimisation. Although this model is not expensive to evaluate we resorted to building a surrogate model, which allowed us to use an efficient target optimisation algorithm we have demonstrated earlier [11,26]. Target optimisation is significantly easier compared to global optimisation as the optimiser is allowed to stop after finding only few conditions that satisfy a target, compared to the problem of finding a global optimal.

The target functions and their corresponding values in the optimisation presented below were the yield, *y*, of **2** defined in Equation 1, with a desired value of 100%, and a specific cost function given in Equation 2 with a target value of 2,108 £ h kg^−1^. This cost function was selected to account for the material and energy consumption, and the reaction time with respect to the amount of product **2**. Thus, *cost*_el_ and *cost*_i_ represent the electricity and material costs, whereas *W*_el_ and *m*_i,0_ denote the consumed electricity and materials, respectively. The product output 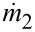 with

[3]
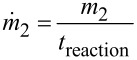


combines the amount of product **2** with the necessary reaction time. The cost target value was derived based on a reaction with the shortest reaction time and highest yield, identified from a series of prior experiments (see Table S2 and Figure S5 in Supporting Information File 1 for further details) [18].

[1]
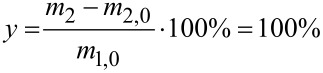


[2]
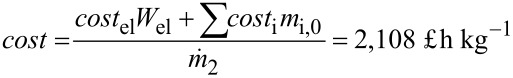


The surrogate model was trained on the 64 experimental points obtained for model parameter identification. In case of an unknown mechanism, the experiments for mechanism discovery could also be included in the training set, which leads to a data-efficient approach. However, they might not be the most informative for training the surrogate. The output of the surrogate model is the suggested next experiment to perform, which was used as an input to the process model. Upon reaching the targets in silico after a number of optimisation iterations, the successful input conditions were verified experimentally, to confirm the predictions.

**Figure 4:**
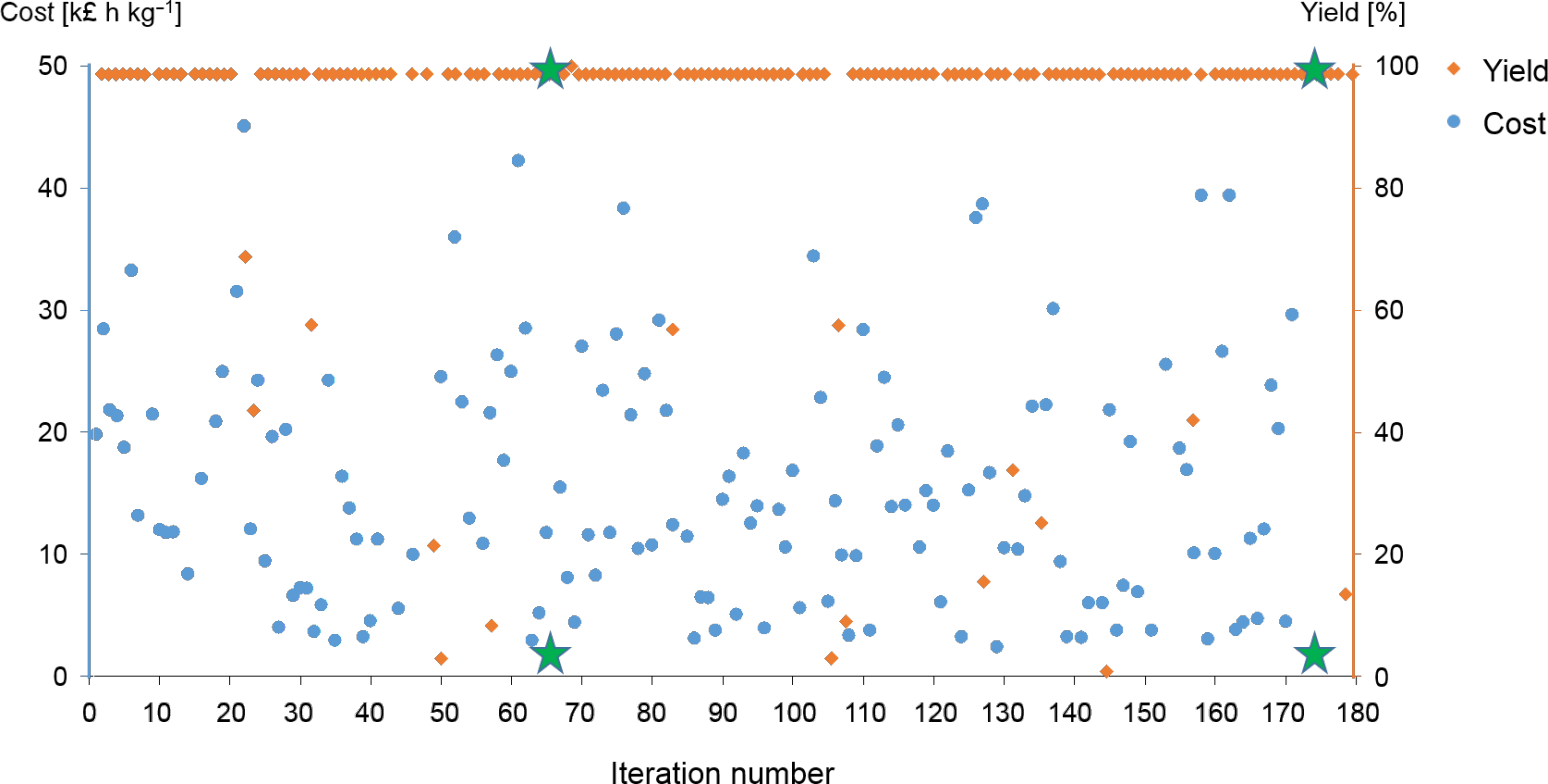
Results of in silico iterations of the multi-objective active learner (MOAL) algorithm [26]. Each iteration produces two resulting values, one for yield and one for the cost function. Targets were 100% for yield and 2,108 k£ h kg^−1^ for the cost function. Green stars signify experiments that satisfy the selected targets.

The in silico results for the optimisation target cost and yields are shown in Figure 4 and Table 4. It can be seen that out of 174 iterations, several points were very close to the targets and two optimal sets of conditions satisfy both targets (these iterations are marked with stars). The simulation results of the two identified successful sets of conditions both predict a yield of 98.72%. The experimentally obtained yields in the validation of the two sets of conditions were determined to be >99%, which is caused by the uncertainty of the applied GC method including sample preparation, which lead to ±1% variance in the yield value. The algorithm is not expected to exhibit fast convergence, since it is exploring the reaction space to develop a better statistical model. The physical experiments performed thereafter confirmed prediction of the successfully attained target values. Hence, only the successful predicted experimental conditions *X*_opt_ were tested in real experiments, which saved time, cost and material, otherwise associated with testing false predicted reactions.

**Table 4:** Experimental conditions and results of the experimental validation of the two successful predictions that met the target specifications.^a^

Iteration	*T* [ºC]	*t*_reaction_ [min]	R_acid-1_	R_cat-1_	Yield [%]	Cost [k£ h kg^−1^]

66	107	9	46.1	0.077	>99 (98.72)	1.79 (1.92)
174	101	10	41.4	0.077	>99 (98.72)	1.79 (1.93)

^a^R_acid-1_: ratio of the concentrations of acetic acid and compound **1**, R_cat-1_: ratio of the concentrations of catalyst and compound **1**. Values in brackets are the predicted values by the physical model. Further information regarding the experimental conditions is given in Supporting Information File 1, Table S6.

The cost target was more difficult to reach than the yield target, which was already fulfilled after the first iteration and later for most of the proposed experiments. This can be seen by the large fluctuation in the cost values for the proposed experiments over the 174 iterations. One possible reason might be the structure of the cost function with many input variables and strong sensitivity with regards to product amount and reaction time. The reaction conditions shown in Table 4 indicate relatively similar conditions with respect to temperature, reaction time as well as acid and catalyst loading, and do not at this stage demonstrate a case of multimodality.

**Figure 5:**
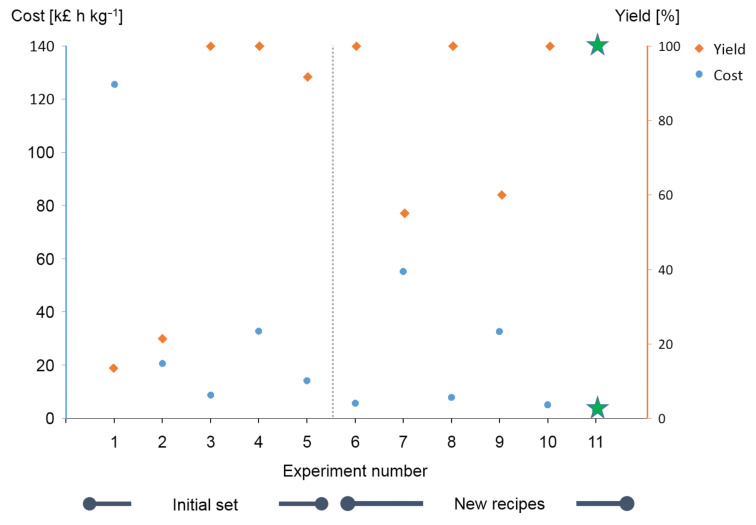
Results of the optimisation driven by a statistical algorithm and in the absence of a physical process model. Results of the training set and of sequential optimisation are shown. Information regarding the experimental conditions is given in Supporting Information File 1, Table S7.

We have also applied the same target optimisation algorithm for direct improvement of this chemical system as a ‘black-box’ sequential optimisation. For this approach five experiments were used as a training set, using Latin Hypercube space filling algorithm; the results are shown in Supporting Information File 1, Table S7 (Expt. 1–5). Figure 5 shows results of the initial set of experiments on the left side of the plot. It is noted that two of the five training experiments did incidentally meet the target value for yield at the conditions set. All outputs, regardless whether they reached the desired target values, were included into the training set and the algorithm was re-trained on the updated set once more. This iterative process was conducted six times. After a single iteration, the results of the first suggested set of conditions were already more promising than any of the training points. The target for yield has been met, as perhaps expected as it was optimal for two of the training examples as well, and the value for cost has been significantly reduced, getting closer to the pre-defined target. Whilst it is observed that experiments 7 and 9 have a large margin of error with regards to the targets, this is due to the exploration function of the algorithm.

Four of the new suggested experimental conditions achieved high yields with the accepted accuracy and had lower cost scores than even the lowest that was found in the initial set. The recipe at the 6th and final iteration following the training set corresponds to a temperature of 102 ºC, reaction time of 15 minutes, acid–substrate ratio of 27.85 and catalyst–substrate ratio of 0.084. With these final conditions, the algorithm converged as both targets were met simultaneously for the first time.

### Comparison of the two optimisation approaches

In this work we used the automated flow set-up combined with MBDoE approach to rapidly develop a good-enough process model, which was then used to train a surrogate model and perform a target optimisation. This resulted in two new sets of reaction conditions which both provided better results than the ones obtained previously. In our second approach we used the experimental flow system as a ‘black-box’ and employed the same statistical target optimisation algorithm to experimentally find the conditions that satisfy the set targets. In this specific case the ‘black-box’ target optimisation is extremely efficient and found suitable reaction conditions within a very small number of experiments. However, no knowledge about the system was generated. The approach of using automated flow experiments in combination with MBDoE allows to minimise experimental effort compared to classical kinetic studies, but results in a process model that can be directly used in optimisation. This approach is clearly preferred for the cases when a model structure could be identified. There would be many practical cases when due to complexity of chemistry it would be unrealistic to develop a physical model within a reasonable timescale. Then the ‘black-box’ approach is a viable option.

## Conclusion

In conclusion, we present an approach of using model-based design of experiments, based on the first principles model structure, in automated flow experiments, and coupling of the process models with a statistical machine learning based target optimisation. We demonstrate that MBDoE offers a significant potential for efficient and rapid generation of process models in flow experiments. The developed process model enables in silico training of the optimisation surrogate model and cost effective determination of process conditions that satisfy the set performance targets. While this is certainly faster than physical experiments, we also show that the self-optimisation works well when trained on a space-filling method to avoid many necessary experiments for model generation. This results in a set of experiments that reach the pre-defined targets in six iterations, although it does not provide any process knowledge. Hence, a combined approach, leading to generation of a surrogate model and a physical model has unique advantages of rapid optimisation and simultaneous generation of process knowledge.

## Experimental

### Reaction system and analysis

All reactions were performed in continuous segmented flow using the R2^+^/R4 system by Vapourtec, see Figure 2. The reaction mixture segments and the solvent were pumped through a 10 mL polytetrafluoroethylene (PTFE) tubular reactor and quenched in an ice bath at the reactor outlet. A minimum segment volume of 2 mL was found to be necessary to avoid dispersion effects in the centre of the segment. The segments were detected using the in-line UV cell, which allowed automatic triggering of the GC (Agilent 6850) to sample the segment at its centre. The flow GC vial was designed by Daniel Geier and Ralf Thelen from the Institut für Technische und Makromolekulare Chemie (ITMC) at RWTH Aachen University and manufactured in-house in Cambridge. GC analysis was performed for product **2** with an accuracy of ±0.0005 mol L^−1^. Due to the decomposition of **1∙**HOAc, **B** and **A** to **1** during sampling following a reaction, the reaction mixture was analysed for species **1** prior to beginning a reaction, with an accuracy of ±0.0003 mol L^−1^. All communication between instruments was custom-coded in LabVIEW and communication with Vapourtec was via its proprietary Excel interface. Further details of the set-up and the on-line auto-sampling strategy, as well as a protocol for sample preparation and experiment execution are provided in Supporting Information File 1.

#### Materials

Toluene (Sigma-Aldrich, anhydrous, 99.8%), acetic acid (Sigma-Aldrich, ReagentPlus, ≥99.0%), acetic anhydride (Sigma-Aldrich, ReagentPlus, ≥99.0%), 1,1,2,2-tetrachloroethane (Sigma-Aldrich, reagent grade, ≥98.0%), palladium(II) acetate (Sigma-Aldrich, reagent grade, 98.0%, no further purification steps were applied, the same batch was used for all experiments, stored according to manufacturer’s suggestions), (diacetoxyiodo)benzene (Sigma-Aldrich, ≥98.0%) were all used as received. 3,3,5,5-Tetramethylmorpholin-2-one was synthesised as described elsewhere [18].

#### Model development and analysis

A process model was developed on the basis of the previously discussed reaction mechanism and DFT estimates of the rate constants (see Table S1, Supporting Information File 1) [18]. The kinetic model was developed as a well-stirred tank reactor. The model includes kinetic equations, energy and material balances as well as constitutive equations. A lumped model was created as each reaction segment was assumed to be ideally mixed; thus no excess volume was considered for mixing. As the reaction takes place in a homogeneous liquid phase, and as the tube dimensions are small, there was no need to account for mass transfer effects. For simplicity, the slightly endothermic nature and hence the heat of reaction for the C–H activation was neglected.

The temperature effect in the reaction steps shown in Scheme 2 were expressed using the Arrhenius equation in its re-parametrised form, shown in Equation 4 and Equation 5 [27–28]. This facilitates subsequent parameter estimation by decoupling the kinetic parameters of each reaction. Assuming equilibrium for the three reversible reaction steps in Scheme 2, the 8 kinetic parameters of interest in this reaction sequence were the reference reaction rate constants (*k*_j,ref_) and activation energies (*E*_a,j_), where *j* 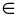 {0, 1, 2, 3,} given by Equation 4 and Equation 5).

[4]
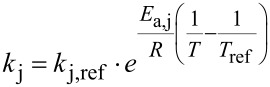


[5]
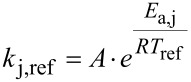


The temperature-dependent volumetric reaction rates 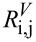 of compound *i* in the reaction *j* were modelled by Equation 6, in which *ν*_i,j_ are the stoichiometric coefficients of a compound *i* in the reaction *j*, *c*_i_ denotes the molar concentration of the compound *i*, *k*_j_ represents the reaction rate constant of the reaction *j* and *n*_i,j_ gives the order of the reaction. All reaction steps in Scheme 2 were found to be first order with respect of the participating compounds, except for the oxidant PhI(OAc)_2_ which is of zero-order dependency [21].

[6]
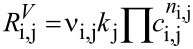


In addition, the overall and the component mole balances, Equation 7 and Equation 8, were written for the process model, where *V* denotes the reaction volume.

[7]
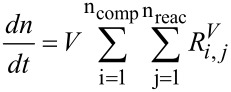


[8]
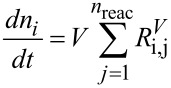


The balances were constructed for a single reaction mixture segment, which was assumed to behave as a batch reactor, as samples were taken in the dispersion-free centre of the segment.

For the purpose of calculating the cost associated with heating the system, a steady state energy balance, Equation 9, was established.

[9]
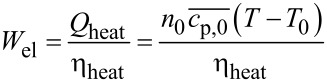


were η_heat_ denotes an overall efficiency of conversion of electrical into thermal energy of the reaction mixture segment. This efficiency was determined experimentally for the employed reactor system by measuring the electrical power input to the Vapourtec heating system needed to increase the temperature of a reaction mixture stream with a set flowrate and of known composition, thus with known molar flow and heat capacity, from ambient temperature of approx. 20 °C to a reaction temperature of 70 °C. By inserting these values into Equation 10, the value for the energy-conversion efficiency was calculated and kept constant.

[10]
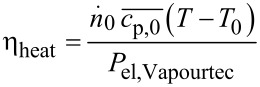


To complete the process model, simple constitutive equations were applied and initial parameter values were computed. The latter were identified based on Gibbs free energies of reaction for the chemical system, which were obtained from a priori DFT calculations with an accuracy of ±7 kJ mol^−1^ [18]. These values were related to kinetic parameters through the exponential van‘t Hoff equation.

Subsequently, the model was investigated and tested for identifiability to ensure its structural soundness, i.e., that it can be used to uniquely determine its parameters. This was done using an established method, detailed elsewhere [29–30]. It is worth noting that structural identifiability is tested under the assumption of noise-free measurement data and no uncertainty of the model. Thus, it does not necessarily imply practical identifiability as the measurement data for the parameter estimation is usually superimposed by noise and errors [31]. Thereafter, an estimability analysis, based on visual inspection of the local dynamic sensitivity curves [32], confirmed that all parameters except those for reaction with *j* = 1 can be determined with sufficient accuracy. For this reaction step, low sensitivity of the measurable quantity, concentration of compound **2**, was found (see Supporting Information File 1, Figure S9 for further details). The local dynamic sensitivity curves of the remaining parameters were used to identify the time intervals with the maximum sensitivity of the parameters (see Supporting Information File 1, Figure S9), indicating reasonable sample points to obtain sensible measurement data for parameter estimation.

The physical model was implemented in gPROMS. The D-optimality criterion was selected to refine the model using the model-based design of experiments (MBDoE) and parameter estimation suite of gPROMS by employing standard solver settings. Further details on this procedure are given in Supporting Information File 1 together with constraints employed for the experiment control variables in the MBDoE optimisation problem (Equations S8–S21, Supporting Information File 1).

The experimental design and parameter estimation strategy included two steps. In the first step, experiments were designed at a reference temperature *T*_ref_ = 70 ºC to eliminate *E*_a,j_ as a parameter in each reaction *j*, see Equation 4 and Equation 5). A t-test was used as a statistical method for judging the increase in precision of the predicted parameter and, hence, the statistical significance of the estimates which is attained if the predicted t-value exceeds a reference value *t*_ref_. The performed experiments would therefore generate data to enable estimation of the reference reaction rate constants *k*_j,ref_ independent of *E*_a,j_. After these parameters have been determined to sufficient accuracy, they were kept constant and experiments were designed at temperatures different from *T*_ref_ in the second step to obtain data for the subsequent estimation of *E*_a,j_. The combination of MBDoE and subsequent parameter estimation was repeated twice in both steps to increase parameter precision, whilst keeping the experimental effort low. This was necessary to ensure good parameter improvement in the case of poor initial parameter guesses.

#### Algorithm for statistical optimisation

One key element of the proposed framework for self-optimisation of reaction conditions is the statistical multi-target optimisation method. For this purpose, the multi-objective active learner (MOAL) algorithm coded in the numerical computing environment MATLAB (v.2015b) was adopted, which combines Gaussian processes as a surrogate model with the concept of mutual information and a genetic algorithm [26]. To apply it to the chemistry under investigation, the algorithm was provided with specified targets *Y*_target_ for the optimisation and defined experiment design variables *X* = [*T, t*_reaction_*, c*_1,0_*, c*_AcOH,0_*, c*_cat,0_] as the degrees of freedom. The latter were bounded by the corresponding constraints (see Supporting Information File 1 for details). A set of 2,000 randomly generated candidate solutions, uniformly distributed within the allowed design space was employed, because the algorithm works with discrete evaluation techniques for the optimisation. The initial training set [*X*_tr_, *Y*_tr_(*X*_tr_)] contained the input variables *X*_tr_ and measurements of the corresponding target values *Y*_tr_(*X*_tr_), which were adopted from the MBDoE approach. It was updated continuously, so that at each iteration of the algorithm, a new training point was added. Binary Gaussian process classification was included into the algorithm to account for feasible and infeasible solutions in *X*, hence learning the promising regions of the design space and evolving some internal process knowledge stepwise with each new iteration. An infeasible solution could occur if an experiment fails in the laboratory. Thus, each point in the training was equipped with one more value, providing information on its feasibility (1) or infeasibility (−1). The current limitation of this approach is that it does not automatically distinguish if the experiment failure identifies the region of design space where the specific reaction is not working, or the failure was due to a random fluke and the same experiment, if repeated, would be successful. There is a way of dealing with this problem, which we will implement, when the algorithm will be published.

After classification of the candidate solutions and training samples, the Gaussian process was trained by fitting the so called hyperparameters of its covariance and likelihood functions by maximising the marginal likelihood with a conjugate gradient optimiser. In this way, a statistical surrogate model was created to provide an approximated response surface for the underlying problem of investigation. This response surface was used to evaluate the feasible candidate solutions and subsequently identify a best solution 
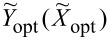
 with corresponding experimental conditions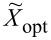. As only discrete candidate solutions were evaluated by this method, the Non-dominated Sorting Genetic Algorithm-II (NSGA-II) was employed for perturbation of 
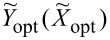
 to explore the neighbourhood for further refinement of the generated solution. The resulting combination of input and output conditions were subsequently assessed against the targets. If the targets were attained within acceptable tolerance, the results were accepted and the statistical algorithm converged. Otherwise, the results were fed back into the training set and a new iteration was started.

#### In silico optimisation

The in silico optimisation process was initiated by first training the MOAL algorithm (with the data generated for the purpose of parameter estimation of the physical model described above). This enables the algorithm to construct a statistical surrogate model and suggest a set of experimental conditions which might give results that are closer to the targets. This set is then fed into the physical model to predict what outputs are expected as though the experiment had been conducted. This process is repeated until the required tolerance is reached. A margin of tolerance was included, such that results within 10 and 1.5% of the target values for cost and yield, respectively, were taken as successful, due to the expected difficulty in achieving those targets. Subsequently, the successful reaction conditions found in silico were tested experimentally. In the case of failure, the experimental results were fed back into the algorithm and the target optimisation loop starts again. Otherwise, the algorithm converged and suitable experimental conditions were identified.

In principle, standard optimisation approaches employing the physical model directly to identify optimum operating conditions could be used, but would give poor results in case of uncertainty and restricted validity of the physical model. However, by applying the MOAL algorithm, technical difficulties regarding multi-objective global optimisation can be overcome. Furthermore, the proposed optimisation procedure can deal with potential uncertainties and restricted validity in the physical model. This is achieved by the machine learning functionalities of the MOAL algorithm, which retrain the algorithm not just on the physical model but also on unsuccessful experiments, erroneously predicted as suitable by the physical model. Thus, it obtains information beyond the capabilities of the physical model. An additional point is, that the MOAL algorithm proved to be especially suited for the detection of multiple possible solutions to indicate multimodality, which is challenging for standard optimisation methods, but can yield valuable information in the current case.

#### Statistical closed-loop optimisation

Statistical target optimisation was performed using the MOAL algorithm. Latin hypercube sampling (LHS) was used to discretise the experimental space initially. An overview of this sampling strategy is laid out for one variable in Figure S10 in Supporting Information File 1. A uniform distribution was taken for the input variables and hence the cumulative distribution function was linear. In this case k is five and N was the number of initial experiments to be conducted, which was decided to be five. This number of initial training experiments was selected as in the previous application of the MOAL algorithm for laboratory optimisation [11], the same number of training samples was applied for an optimisation of two targets, but with a 14 dimensional design space, instead of five dimensions in the current case. Still, the algorithm converged within 17 iterations (including the five training experiments). Hence, we assumed, that for the current work with less design space dimensions, the algorithm would learn the response surface as fast as it was the case for its previous application.

**Table 5:** Nomenclature.

Symbol	Definition	Units

η_heat_	heat efficiency	–
ν_i,j_	stoichiometric coefficient of component *i* in reaction *j*	–
*A*	pre-exponential factor in Arrhenius equation	case dependent
*cost*	investigated target value	₤ h kg^−1^
*cost*_el_, *cost*_i_	cost of electricity, cost of material component *i*	case dependent
*c_i_*_,0_	initial component concentration	mol L^−1^
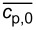	average molar heat capacity	J mol^−1^ K^−1^
*E*_a,j_	activation energy of reaction *j*	J mol^−1^
*k*_j,ref_	reference rate of reaction in reaction *j*	case dependent
*m*_i_, *m*_i,0_	mass of component *i*, initial mass of component *i*	kg s^−1^
*n*_i_, *n*_0_	number of moles of component *i*, total number of moles initially	mol
*P*_el,Vapoutec_	electrical power uptake of Vapourtec flow system	W
*Q*_heat_	heat	*J*
*R*	universal gas constant	J mol^−1^ K^−1^
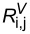	reaction rate of component *i* in reaction *j*	mol L^−1^ s^−1^
*t*, *t*_reaction_	time, reaction time	s, min
*T*, *T*_ref_, *T*_0_	temperature, reference temperature, initial temperature	°C
*V*	volume of system	L
*W*_el_	electrical work	J
*X*	experiment design variables	–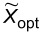	proposed suboptimal inputs	–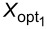	proposed optimal inputs	–
*X*_tr_	input training matrix	–
*y*	yield	%
*Y*_target_	target outputs matrix	–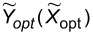	proposed suboptimal outputs	–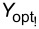	proposed optimal outputs	–
*Y*_tr_	output training matrix	–

## Supporting Information

File 1 Details of experimental set-up and protocols, table of a priori data taken from our previous study, details of model development, MBDoE results, and LHS results.
