# Supplementary material for: Self-optimisation and model-based design of experiments for developing a C–H activation flow process
Source: Beilstein J Org Chem. 2017 Jan 24;13:150–63. doi: 10.3762/bjoc.13.18 (PMC5301945; doi:10.3762/bjoc.13.18)
Supplement: File 1 — Details of experimental set-up and protocols, table of a priori data taken from our previous study, details of model development, MBDoE results, and LHS results. [file Beilstein_J_Org_Chem-13-150-s001.pdf]

Supporting Information

for

Self-optimisation and model-based design of experiments for developing a C–H activation flow process

Alexander Echtermeyer[1,2], Yehia Amar[2], Jacek Zakrzewski[2] and Alexei Lapkin[2,*,§]

Address: [1]Aachener Verfahrenstechnik – Process Systems Engineering, RWTH Aachen University, Aachen, Germany and [2]Department of Chemical Engineering and Biotechnology, University of Cambridge, Cambridge, United Kingdom

*Corresponding author

Email: Alexei Lapkin- aal35@cam.ac.uk

§+ 44 1223 334796

Details of experimental set-up and protocols, table of a priori data taken from our previous study, details of model development, MBDoE results, and LHS results

A priori data taken from previous DFT study

Table S1 shows data from our group's earlier DFT study [1], which was used in this work to obtain initial guesses for reaction parameters. This data was regarded as a priori chemical knowledge.

Table S1: Gibbs free energy values obtained from DFT calculations, and their corresponding kinetic parameters.

Parameter	Gibbs free energy [J mol^{-1}]
$K_{c,\,0,\,-0}$	95,978
$K_{c,\,1,\,-1}$	−42,090
$K_{c,\,2,\,-2}$	−25,845
$k_{3,\,ref}$	−3,153

$K_{c,j,-j}$ is equilibrium constant of reaction j, $k_{3,ref}$ is the reference rate of the reaction 3.

Experimental

Protocol for sample preparation

- The required mass of the starting material was carefully pipetted into a 10 mL beaker (or a 30 mL Erlenmeyer flask for experiments requiring larger amounts) and prevented from exposure to air using a rubber stopper.
- The required mass of the catalyst was weighed onto a small strip of paper using a spatula and fed into the beaker, followed by a similar procedure for the required mass of oxidant.
- The required volume of toluene was added using a 10 mL measuring cylinder and an Eppendorf pipette. Subsequently, the required volume of acetic anhydride was added using an Eppendorf pipette, followed by a similar step for the required volume of acetic acid and similarly for the GC internal standard, 1,1,2,2-tetrachoroethane, whereby the latter was done in a fume hood.
- Following, the sample preparation, it was stirred for up to 30 minutes until the oxidant dissolved fully and 1 mL of the mixture was analysed in the GC to characterize the sample at the initial time.

Reactor setup

For the physical experiments presented in this study a $R2^{+}$/R4 automated reactor system by Vapourtec was employed. The $R2^{+}$ pump module was driven by two integrated high performance pumps with adjustable flow rates between 0.001 and 10 ml/min at a system pressure of up to 10 bar. The R4 reactor module was set up in a flow tube arrangement and included an air heater enabling temperature control up to 150 ºC. Furthermore, it was fitted with a pair of coiled injection loops (2 mL each) positioned immediately following the pumps. A segmented flow operation mode as applied, meaning that for each reaction a total segment volume of 2 mL was sent into the reactor. A coiled tube reactor with a total volume of 10 mL was used. A back-pressure regulator of 7 bar was fitted and applied in-line to establish sufficiently high system pressure for stable pump operation and prevent on gas formation in the reaction system which impacted subsequent segment detection.

Analytical setup

For the present work, an Agilent 6850 Network GC, equipped with an automated liquid sampler (ALS), was employed. The column, coated with the stationary phase, was a HP-1 30 m × 0.32 mm × 0.25 µL column. Helium was used as the carrier gas (2.271 mL min^{-1} and 12.748 psi) and air as the make-up gas. The samples were analysed using auto injection with

an injection volume of 1 μL, split mode and a split ratio of 200:1. The initial temperature of 100 °C was held for 1 minute and then ramped at 30 °C min^{-1} to 250 °C with a hold time of 1 minute, resulting in an overall method time of 7 minutes.

To enable on-line analysis of composition data by GC, a special ALS carousel was applied to connect the analytical device to the continuous flow system and is shown in Figure S1 and S2. The incoming stream was connected to one of the carousel slots and sent to a specially designed sampling chamber, capped with a septum. The stream entered the chamber as a small fountain in the middle from which a sample was taken through the septum by the ALS. The exiting stream was subsequently sent to a waste beaker.

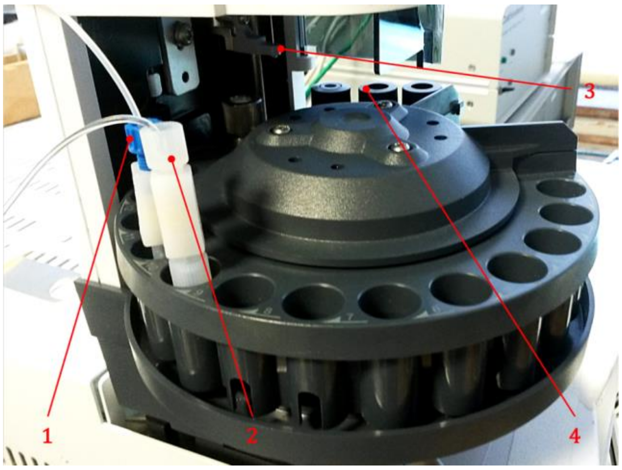

Figure S1: Image of the GC carousel. 1: inlet of reaction mixture from automated reactor system, 2: outlet of reaction mixture, 3: syringe from automatic liquid sampler (ALS), 4: vials of solvent to clean the ALS syringe and waste vial.

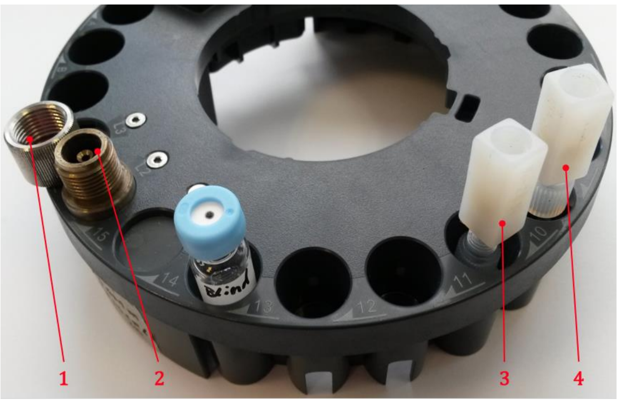

Figure S2. Image of the GC carousel. 1: cap with a septum to seal the sampling chamber, 2: sampling chamber itself, 3: inlet of pipes carrying reaction mixtures from the automated reactor system to the GC, 4: outlet leading to waste beakers following GC analysis.

GC calibration curve

A calibration curve for the GC analysis, using an internal standard (IS), was established for the product **2** in the upper plot and for the starting material **1** in the lower plot, shown below in Figure S3. The calibration curve Equations S1 and S2 are shown as well, where A_i with i ∈ {**1**, **2**, IS} corresponds to the Area beneath the GC curve for the specific component.

$$c_2 = \frac{\frac{A_2}{A_{IS}} + 0.0759}{1.8743} * c_{IS} \tag{S1}$$

$$c_1 = \frac{\frac{A_1}{A_{IS}} + 0.1544}{2.4342} * c_{IS} \tag{S2}$$

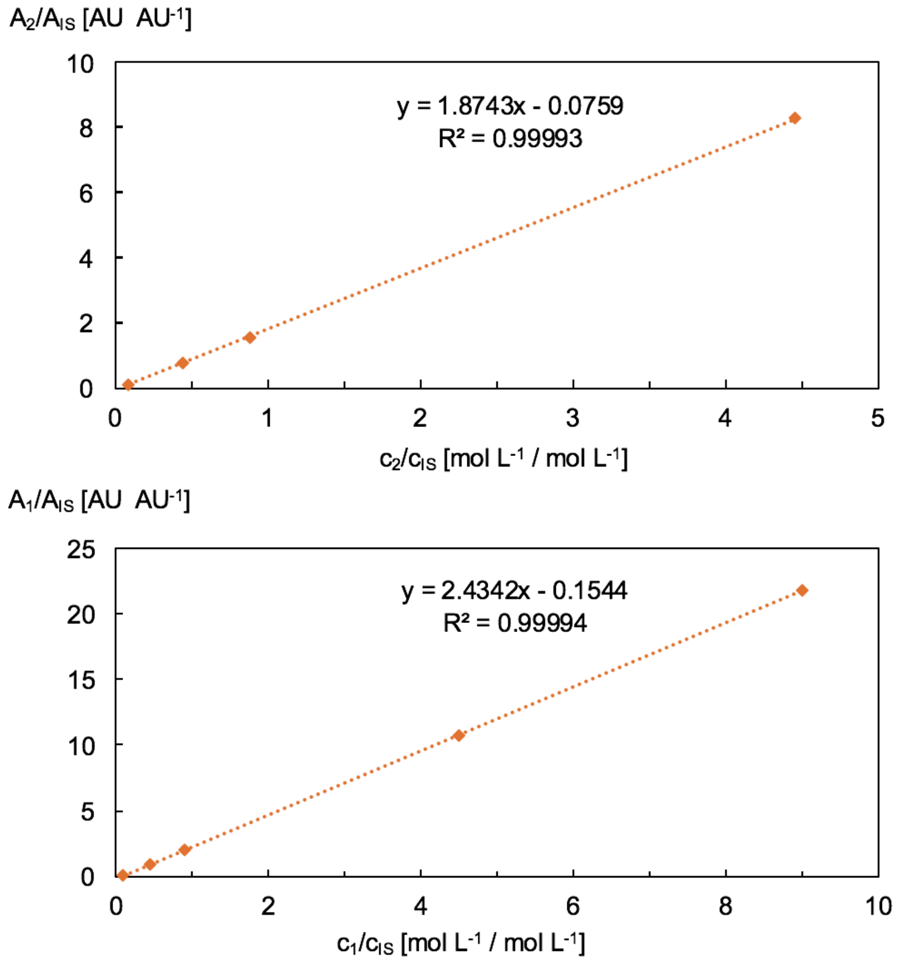

Figure S3: Calibration curve for GC analysis. Top: product calibration curve. Bottom: substrate calibration curve. AZ: product **2**, SM: starting material **1**, IS: internal standard, AU: arbitrary unit, A: Area, c: concentration.

Figure S4 shows the variance in the measured product concentration for the GC calibration curve, enabling parameters for a linear variance model regarding that concentration to be found via coefficient comparison of the model and the regression curve shown below.

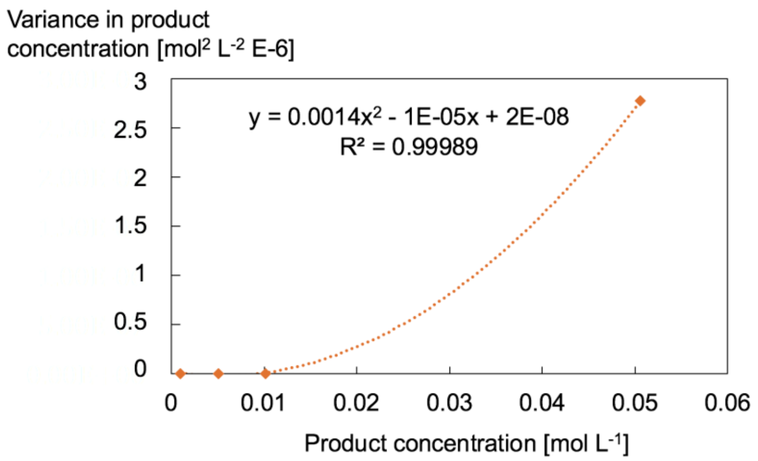

Figure S4: Regression curve of variance in the product concentration for the GC calibration curve. Parameters for a linear variance model regarding that concentration were obtained by comparing coefficients of the model and this graph.

Determination of targets

The experimental conditions and results for the experiment that served as the basis for the targets for the multi-objective optimisation are shown in Table S2 and Figure S5.

Table S2: Recipe for an experiment conducted by Zakrzewski et al. [1], which served as the basis of the target determination for cost.

T [°C]	c_{ox} [$mol\ L^{-1}$]	$c_{1,0}$ [$mol\ L^{-1}$]	$c_{AcOH,0}$ [$mol\ L^{-1}$]	$c_{cat,0}$ [$mol\ L^{-1}$]	$c_{anhydride}$ [$mol\ L^{-1}$]
90	0.078	0.05	0.58	0.005	0.21

1: substrate, AcOH: acetic acid, cat: catalyst.

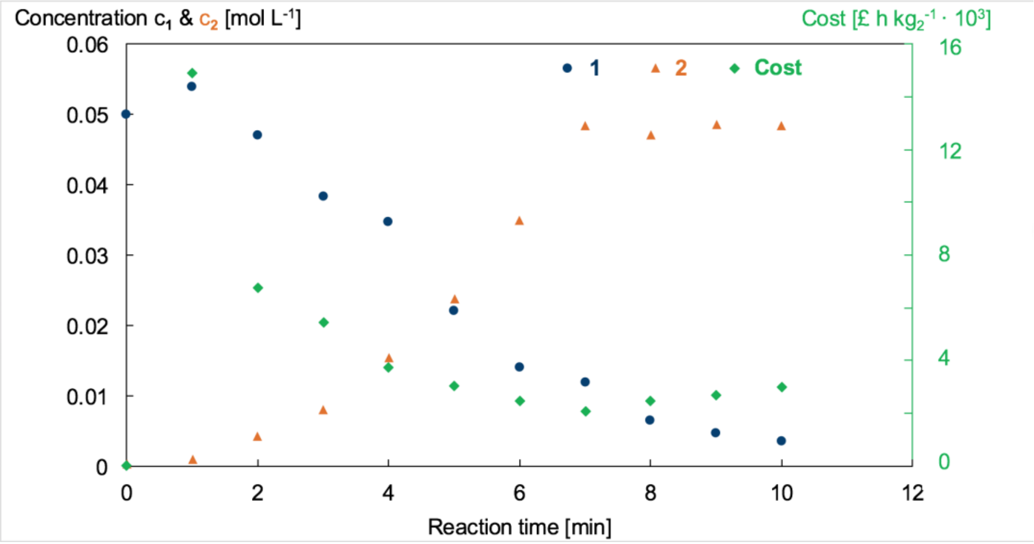

Figure S5: Experimental results for the experiment by Zakrzewski et al. [1], which served as the basis of the target value determination for cost. **1**: substrate, **2**: product.

Experiments to determine solubility of the oxidant

The solubility of the oxidant was needed a constraint on the concentration of the substrate. In Figure S6, the acetic acid concentration of the reaction mixture is expressed over the maximum concentration of dissoluble oxidant at room temperature in a stirred beaker of toluene. On the right hand side, the images show the effect of acetic acid addition. Whilst the upper picture contains no acetic acid, the bottom one contains acetic acid and hence the oxidant has dissolved completely in the solution. A correlation has thus been established (S3) and is used as a constraint for maximum allowable oxidant concentration.

$$c_{ox,solub}\left(\frac{mol}{L}\right) = \frac{c_{AcOH,0}+1.2872}{34.441} \qquad \text{(S3)}$$

Completely dissolved oxidant is important for two reasons. Firstly, oxidant crystals could damage the valves of the Vapourtec pump module, could cause blockages with resulting damage to the tube and pressure warning of the system. Secondly, catalyst is included and accumulated in the oxidant crystals which makes it impossible to relate the measured reaction rates to the real catalyst concentration participating in the reaction.

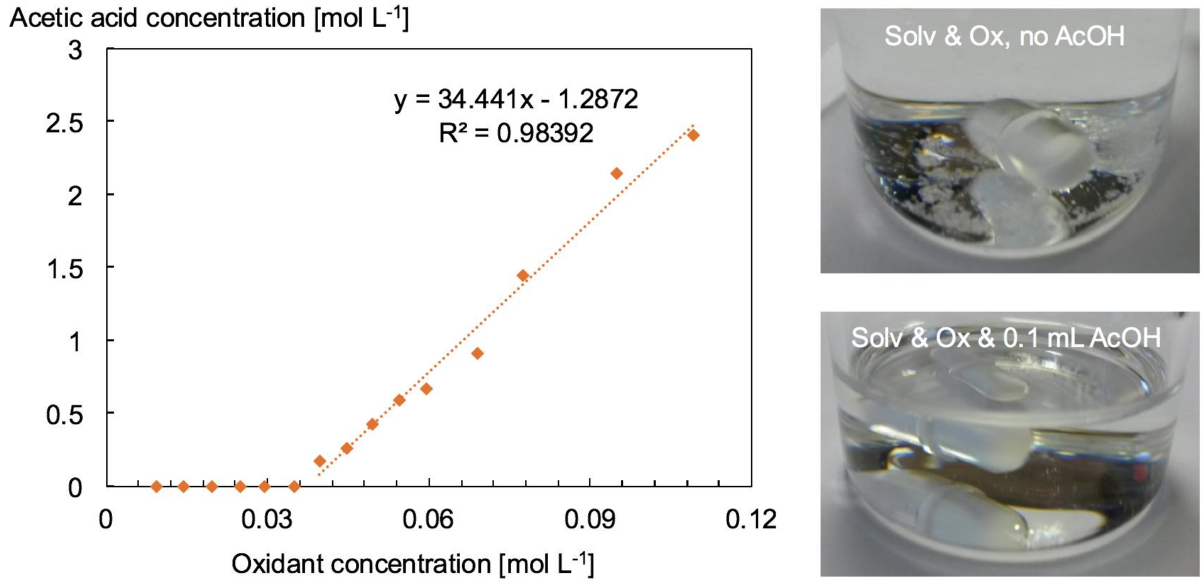

Figure S6: Results of solubility study of oxidant in reaction mixture. This served as a constraint so that the concentration of oxidant does not exceed that which enables it to dissolve in toluene and acetic acid.

Experimental protocol

The reaction mixture was prepared according to the sample preparation section described above (one mixture for one experimental sequence, so that the same mixture was used for all experiments regarding the determination of e.g. $k_{2,ref}$). Initial samples were taken by the GC to characterize the reaction mixture. Thereafter, two injection loops leading into the pumping module were filled with the 2 mL of the reaction mixture, each. The pumping and reactor module were purged of gas, started, flushed with the solvent and the tube reactor was heated to reach the required operation temperature. The LabVIEW control interface and UV detector cell were initialized and the sequence of reaction conditions was loaded into the system. Subsequently, the reaction sequence was started, monitored and controlled via the LabVIEW interface. A reaction mixture segment with a total volume of 2 mL was injected into the system from the injection loops at a predefined flow rate corresponding to the desired reactor retention time. The necessary segment volume was determined based on dispersion measurements and selected to a value of 2 mL to avoid dispersion effects in the centre of the segment, which ensured reliable reaction conditions. Upon detection by the UV cell in-line behind the reactor, the GC sampling by ALS was triggered and a sample was taken from the centre of the segment. Following the sampling, GC analysis was conducted and a new reaction mixture segment was injected from the injection loops. A manual refill of the injection loops with reaction mixture was necessary after two experiments. This procedure

continued until all experiments of one sequence (e.g. 5 experiments for the determination of $k_{2,ref}$) were finished.

The sample analysis file from ChemStation by Agilent was used together with calibration data to relate the GC measurements to product and starting material concentrations. These were employed for the calculation of the target quantity values (cost and yield) for each experiment.

On-line auto-sampling

The hardware for GC on-line auto-sampling is described in a prior section. This section describes aspects regarding the initiation of sampling caused by a trigger signal send from a LabVIEW interface to the GC.

Depending on the concentration of the catalyst $Pd(OAc)_2$, the reaction mixture is coloured deep yellow and therefore a reaction segment can be detected by UV transmission measurements. Figure S7 shows a UV signal for a 2 mL reaction segment in the flow setup (orange curve). When the reaction segment reaches the in-line UV flow cell (at about 20 min in Figure S7), it causes a voltage signal, which is processed in a custom-made LabVIEW interface. To verify a reaction segment in the UV flow cell, the voltage signal is evaluated via four different criteria, which are shown in Figure S7 and are described in the following.

In the case of a detected voltage increase, the interface first checks whether the minimum possible time between two GC measurements, determined based on GC method time and time for cooling down to starting temperature, is elapsed. This criterion is represented by the time threshold Δt in Figure S7 and was determined to be 8 min in the present work. Thus, sampling could only be triggered if the GC would be ready for a new measurement.

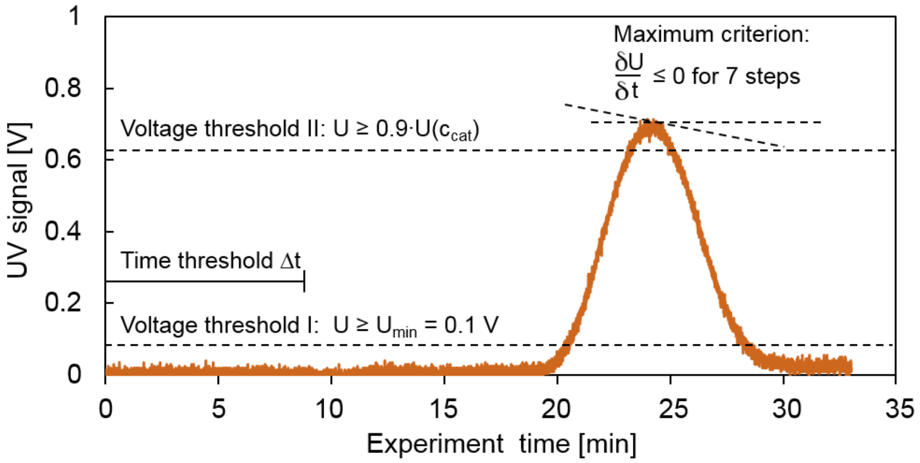

Figure S7: Processing of the UV signal and triggering of the GC ALS based on different criteria which must be fulfilled.

After the Δt-criterion is fulfilled, the voltage signal is further analysed and has to reach two different voltage thresholds to verify a reaction segment in the UV flow cell. Voltage threshold I was set to a value of 0.1 V determined empirically to avoid triggering due to voltage peeks caused by air bubbles in the system or measurement noise.

Voltage threshold II depends on the catalyst concentration as different catalyst loads cause different colour of the segment and hence a different maximum voltage. The dependence between catalyst concentration and voltage signal was assumed to be linear and obtained from a simple calibration using Lambert-Beer law. A value of 90% maximum voltage signal was also determined empirically and is sufficient to identify the dispersion-free zone in the middle of the segment (maximum voltage). It is worth noting, that also a minimum catalyst concentration exists as the UV flow cell has a certain sensitivity. The minimum catalyst concentration in the present work was determined to 0.0002 mol L^{-1} and directly contributes as a hardware constrained in the MBDoE problem further described in a later section.

The fourth and last criterion checked by the LabVIEW interface is the maximum criterion, which assesses the change in the voltage signal. If it is equal to zero or negative for a specific amount of time, the maximum voltage signal and hence the centre of the reaction segment (lowest dispersion and maximum catalyst concentration) was detected in the UV cell.

Only if all four criteria are fulfilled, a trigger signal is consecutively send to the GC ALS, but is delayed by the time, which the reaction segment needs to proceed from the UV flow cell

into the GC sampling chamber minus the time needed by the GC to prepare for sampling (needle cleaning, flushing, etc.). The amount of time needed for the segment to react the GC ALS can easily be calculated based on experiment flow rate and tube dimensions. Dispersion was neglected for this calculation in the present work as the distance between UV cell and GC ALS was negligible compared to the total length of the flow system. The GC preparation time was measured for the employed method. A scheme summarising the different steps in the on-line auto-sampling is shown in Figure S8.

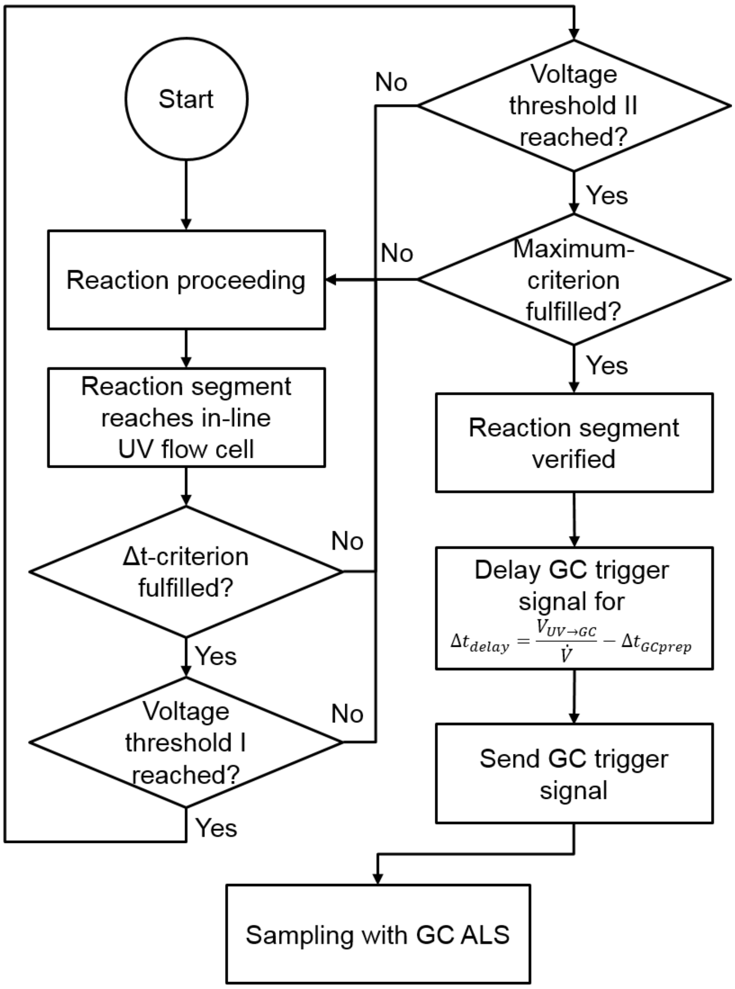

Figure S8: Schematic diagram of on-line auto-sampling process.

Model development

Estimability analysis

In Figure S9, the results of a local sensitivity analysis are shown. This was done for both the reference reaction rate constants and the activation energies using their initial guesses at 70

and 80 ºC, respectively. The other simulation conditions applied for these evaluations are shown in Table S3.

Table S3: Recipe for reaction at which the estimability analysis was performed.

T [°C])	$c_{ox,0}$ [$mol\ L^{-1}$]	$c_{1,0}$ [$mol\ L^{-1}$]	$c_{AcOH,0}$ [$mol\ L^{-1}$]	$c_{cat,0}$ [$mol\ L^{-1}$]	$c_{anhydride}$ [$mol\ L^{-1}$]
70	0.12	0.1	2	0.005	0.2

1: substrate, AcOH: acetic acid, cat: catalyst.

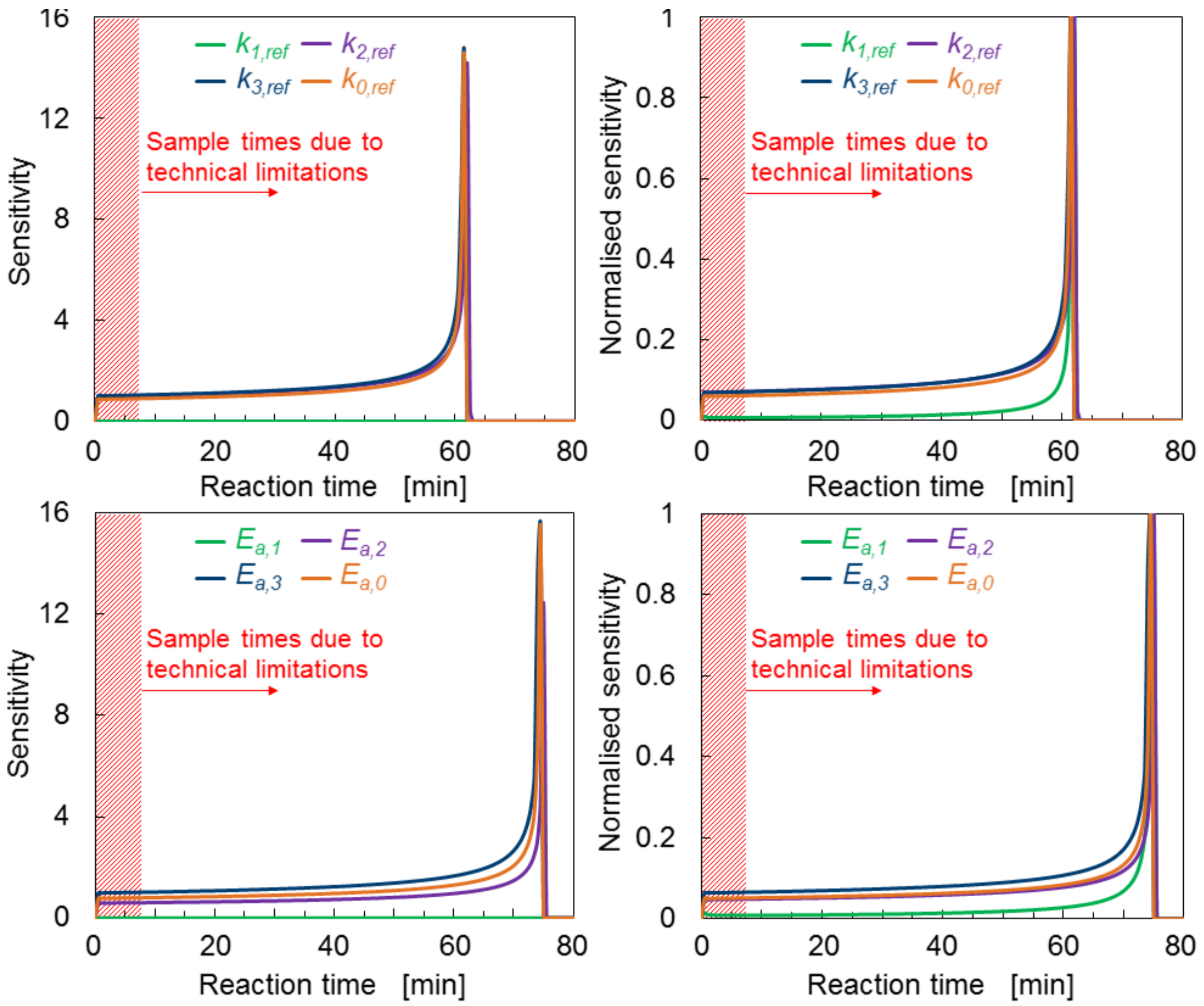

Figure S9: Results for the local sensitivity analysis, which formed part of the estimability analysis. Above: analysis for reference rate constants. Below: analysis for activation energies. It is also shown that $k_{1,ref}$ has no impact on product concentration in the model.

Optimisation problem and constraints on experimental control variables for MBDoE

In such a method, the appropriate values of experimental control variables, φ, such as experimental conditions, measured quantities and sampling times, are selected from the feasible design space Φ. Planned experiments should result in minimization of the variance of model parameters, thus minimizing the parameter confidence region, *i.e.* making the entries of the parameter variance-covariance matrix **V** small. Therefore, some metric of **V** must be minimized as shown in Equation (S4).

$$opt = \min \arg(\mathbf{V}) \tag{S4}$$

By applying the Cramèr-Rao theorem, the inverse of the Fisher Information Matrix (**FIM**), defined by Zullo [2] can be set as a lower bound for **V**. This concept is illustrated by Equation (S5).

$$\mathbf{V} \geq \mathbf{FIM}^{-1} \tag{S5}$$

Thus, the MBDoE problem (9) can be approached by maximizing some metric Ψ of **FIM** to achieve most informative experiments in a statistical sense by employing the experimental control variables φ as degrees of freedom for the optimisation problem, shown in Equation (S6).

Maximize $opt = \max_{\varphi \in \Phi} \Psi(\mathbf{FIM})$

(S6)

s.t. model equations

initial conditions

constraints on φ

Many real valued functions have been suggested as a metric Ψ for evaluating **FIM**. Among the common selections for this, the 'D-optimality' criterion was chosen in the present study. It aims to maximize the determinant of **FIM**, as seen in equation (S7) and is most often applied, owing to its easy geometric interpretation, invariance to parameter scaling and good results when dealing with multiple parameters.

$$D - optimality\text{: } \max\{\det(\mathbf{FIM})\} \tag{S7}$$

The experiment design problem was solved subject to constraints of the design space Φ for φ to ensure safe, economic and physically feasible experiments, given the technical setup and utilized materials.

The following Equations S8–S21 represent the constraints on the feasibility of the reaction mixture recipes and were used as constraints for the experiment control variables in the MBDoE optimisation problem. Some arise from physical limitations of the reactants, such as boiling temperature of the solvent, and some are caused by technical limitations of the experimental setup such as earliest possible sampling time.

$$60\ ℃ \leq T \leq 110\ ℃ \tag{S8}$$

$$413s \leq t_{reaction} \leq 7{,}200 \tag{S9}$$

$$413s \leq t_{sample} \leq 7{,}200 \tag{S10}$$

$$60s \leq \Delta t_{sample} \tag{S11}$$

$$0\ mol\ L^{-1} \leq c_{1,0} \leq 0.1\ mol\ L^{-1} \tag{S12}$$

$$0\ mol\ L^{-1} \leq c_{AcOH,0} \leq 4\ mol\ L^{-1} \tag{S13}$$

$$0.0002\ mol\ L^{-1} \leq c_{cat,0} \leq 0.01\ mol\ L^{-1} \tag{S14}$$

$$c_{oxidant,0} = 1.2 * c_{1,0} \tag{S15}$$

$$c_{anhydride,0} = 0.2\ mol\ L^{-1} \tag{S16}$$

$$c_{IS,0} = 0.01134\ mol\ L^{-1} \tag{S17}$$

$$0 \leq \frac{c_{AcOH,0}}{c_{1,0}} \leq 100 \tag{S18}$$

$$0.05 \leq \frac{c_{cat,0}}{c_{1,0}} \leq 0.2 \tag{S19}$$

$$0 \leq \frac{c_{cat,0}}{c_{ox,solub}} \leq 1 \tag{S20}$$

$$\text{where, } c_{ox,solub}\left(\frac{mol}{L}\right) = \frac{c_{AcOH,0}+1.2872}{34.441} \tag{S21}$$

Results of MBDoE

Tables S4-S5 show the experimental conditions derived from the MBDoE and the corresponding parameters they were used to determine.

Table S4: Results of MBDoE to determine reference reaction rate constants $k_{j,ref}$.

	Experiment 1	Experiment 2	Experiment 3	Experiment 4
Parameter	$k_{0,ref}$	$k_{2,ref}$	$k_{3,ref}$	$k_{0,ref}, k_{2,ref}, k_{3,ref}$
$\boldsymbol{t_{reaction}}$ $[\boldsymbol{min}[$	36	48	10	62
$\boldsymbol{T}$ [°C]	70	70	70	70
$\boldsymbol{c_{1,0}}$ $[\boldsymbol{mol\,L^{-1}}]$	0.0363	0.0459	0.0441	0.0479
$\boldsymbol{c_{AcOH,0}}[\boldsymbol{mol\,L^{-1}}]$	0.2179	1.2007	0.5518	1.8026
$\boldsymbol{c_{cat,0}}[\boldsymbol{mol\,L^{-1}}]$	0.0037	0.0025	0.0088	0.049
$\boldsymbol{n_{samples}}$	7	6	5	11
$\boldsymbol{t-value}$	76.19	23.36	23.36	5.34, 0.03, 6.42
$\boldsymbol{t_{ref}}$	2.92	2.92	2.92	1.94

Table S5: Results of MBDoE to determine the activation energies $E_{a,j}$.

	Experiment 5	Experiment 6	Experiment 7	Experiment 8
Parameter	$E_{a,0}, E_{a,2}, E_{a,3}$	$E_{a,0}, E_{a,2}, E_{a,3}$	$E_{a,0}, E_{a,2}$	$E_{a,0}, E_{a,3}$
$\boldsymbol{t_{reaction}}$ $[\boldsymbol{min}]$	38	55	70	75
$\boldsymbol{T}$ [°C]	61	80	88	88
$\boldsymbol{c_{1,0}}$ $[\boldsymbol{mol\,L^{-1}}]$	0.0372	0.0199	0.0278	0.0193
$\boldsymbol{c_{AcOH,0}}[\boldsymbol{mol\,L^{-1}}]$	3.1626	0.1310	0	0
$\boldsymbol{c_{cat,0}}[\boldsymbol{mol\,L^{-1}}]$	0.0063	0.0019	0.0014	0.001
$\boldsymbol{n_{samples}}$	11	11	10	10
$\boldsymbol{t-value}$	0.05, 0.04, 2.88	0.63, 0.25, 2.33	2.79, 17.1	3.99, 46.8
$\boldsymbol{t_{ref}}$	1.94	1.94	2.02	1.94

Results of in silico optimisation using a priori information

Tables S6 shows the experimental conditions derived from the in silico optimisation employing the MOAL algorithm and using a priori information.

Table S6: Experimental conditions of two successful predictions that met the target specifications during in silico optimisation employing the MOAL algorithm and a priori information.

Expt.	$\boldsymbol{T}$ [°$\boldsymbol{C}$]	$\boldsymbol{t_{reaction}}$ $[\boldsymbol{min}]$	$\boldsymbol{c_{1,0}}$ $[\boldsymbol{mol\,L^{-1}}]$	$\boldsymbol{c_{AcOH,0}}$ $[\boldsymbol{mol\,L^{-1}}]$	$\boldsymbol{c_{cat,0}}$ $[\boldsymbol{mol\,L^{-1}}]$
66	107	9	0.0681	3.1374	0.0053
174	101	10	0.0963	3.9867	0.0074

Latin hypercube sampling

Figure S10 illustrates the concept of Latin hypercube sampling (LHS).

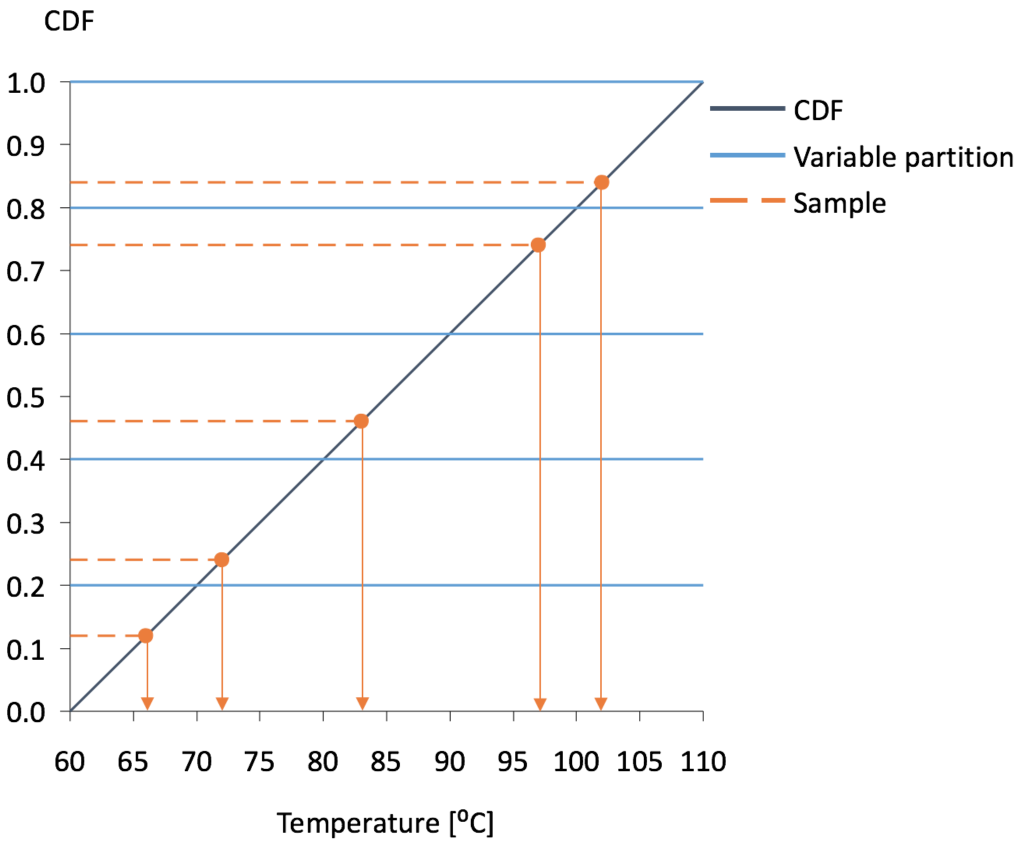

Figure S10: Illustration of Latin hypercube sampling (LHS) approach. A uniform cumulative distribution function (CDF) is taken for each variable and the variable range is partitioned into 5 sections (corresponding to 5 experiments). One random variable value is then taken within each defined range.

The initial set of experiments for the self-optimisation driven by physical experimentation is shown below in Table S7.

Table S7: Recipes for experiments in the optimisation approach driven by physical experimentation.

Expt	T [°C]	$t_{reaction}$ [min]	$c_{1,0}$ [$mol\ L^{-1}$]	$c_{AcOH,0}$ [$mol\ L^{-1}$]	$c_{cat,0}$ [$mol\ L^{-1}$]
**1**[a]	60	58.8	0.0322	1.5435	0.0028
**2**[a]	72	17.0	0.0524	2.7894	0.0051
3[a]	86	41.9	0.0772	2.2206	0.0071

4[a]	97	91.6	0.0181	0.0649	0.0011
5[a]	106	58.8	0.0930	3.5487	0.0089
6[b]	109	30.8	0.0756	2.3368	0.0055
7[b]	109	35.9	0.0057	0.0717	0.0004
8[b]	96	51.1	0.0993	3.1000	0.0047
9[b]	105	115.7	0.0887	3.5923	0.0043
10[b]	86	25.6	0.0998	3.0330	0.0084
11[b]	102	15.3	0.0859	2.3927	0.0072

[a] Recipes obtained by Latin hypercube sampling method, distributed fairly to represent the design space well. [b] Recipes suggested by the optimisation algorithm. **1**: substrate, AcOH: acetic acid, cat: catalyst.

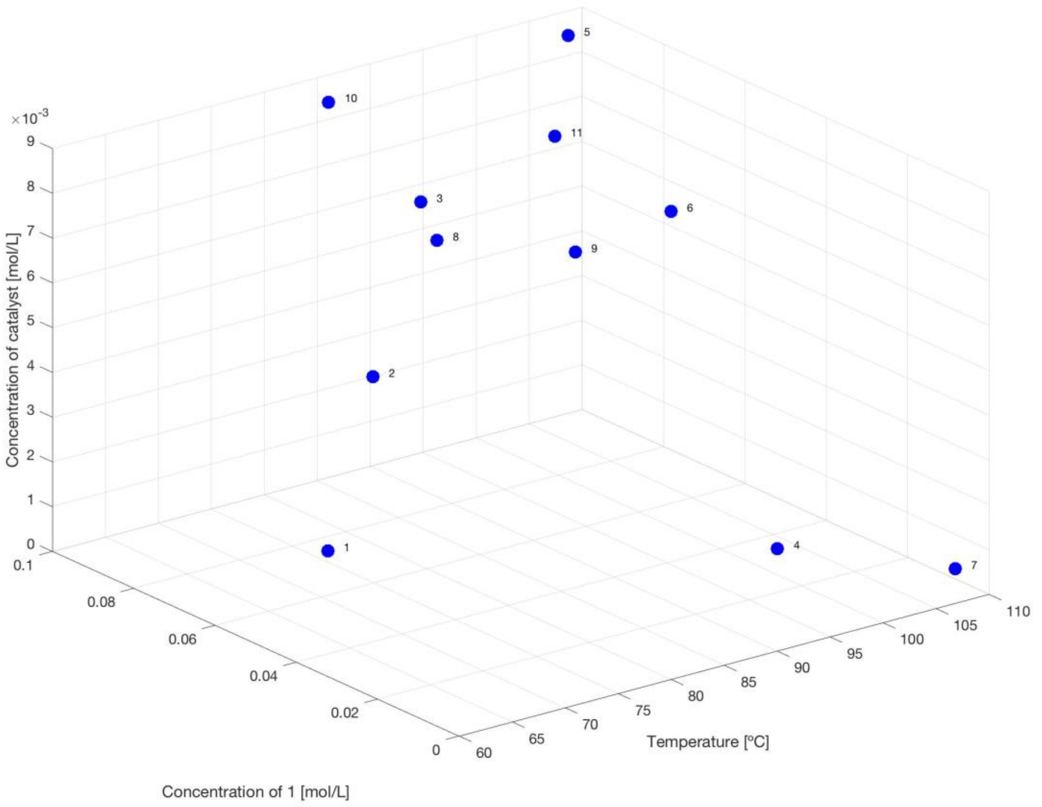

Figure S11: Distribution of recipes from Table S7 regarding temperature, starting material concentration and catalyst concentration. The first five points with grey labels correspond to recepies obtained by a Latin hypercube sampling method, distributed fairly to represent the design space well. Experiments 6–11 are the algorithm-suggested recipes. SM: substrate **1**.

Nomenclature

Symbol	Definition	Units
$\boldsymbol{\eta_{heat}}$	Heat efficiency	-
$\boldsymbol{\nu_{i,j}}$	Stoichiometric coefficient of component *i* in reaction *j*	-
φ	Experiment control variables	Case dependent
Φ	Design space of experiment control variables	Case dependent
$\boldsymbol{A_i}$	GC peak integration result of component *i*	-
Cost	Investigated target value	£ $h\ kg^{-1}$
$\mathbf{cost_{el}}, \mathbf{cost_i}$	Cost of electricity, cost of material component *i*	Case dependent
$c_{i,0}$	Initial component concentration	$mol\ L^{-1}$
$\overline{\mathbf{c_{p,0}}}$	Average molar heat capacity	$J\ mol^{-1}\ K^{-1}$
$\boldsymbol{E_{a,j}}$	Activation energy of reaction *j*	$J\ mol^{-1}$
**FIM**	Fisher Information matrix	-
$\boldsymbol{k_{j,ref}}$	Reference rate of reaction in reaction *j*	Case dependent
$K_{c,j,-j}$	Equilibrium constant of reaction *j*	Case dependent
$\boldsymbol{m_i}, \boldsymbol{m_{i,0}}$	Mass of component *i*, initial mass of component *i*	$kg\ s^{-1}$
$\mathbf{n_i}, \mathbf{n_0}$	Number of moles of component *i*, total number of moles initially	mol
$n_{samples}$	Number of samples	-
$P_{el,Vapoutec}$	Electrical power uptake of Vapourtec flow system	W
$\boldsymbol{Q_{heat}}$	Heat	J
$\boldsymbol{R}$	Universal gas constant	$J\ mol^{-1}K^{-1}$
$\boldsymbol{R_{i,j}^{V}}$	Reaction rate of component *i* in reaction *j*	$mol\ L^{-1}s^{-1}$
$\mathbf{t}$, $t_{reaction}$, Δt_{sample}	Time, reaction time, time between samples	s
$\boldsymbol{T}, \boldsymbol{T_{ref}}, \boldsymbol{T_0}$	Temperature, reference temperature, initial temperature	$°C$
$\boldsymbol{V}$	Volume of system	L
$\mathbf{V}$	Variance-covariance matrix	-
$\mathbf{W_{el}}$	Electrical work	J
X	Experiment design variables	-
$\boldsymbol{\widetilde{X}_{opt}}$	Proposed suboptimal inputs	-
$\boldsymbol{X_{opt_1}}$	Proposed optimal inputs	-
$\mathbf{X_{tr}}$	Input training matrix	-
$\boldsymbol{y}$	Yield	%
$\boldsymbol{Y_{target}}$	Target outputs matrix	-
$\boldsymbol{\widetilde{Y}_{opt}}(\boldsymbol{\widetilde{X}_{opt}})$	Proposed suboptimal outputs	-
$\boldsymbol{Y_{opt_!}}$	Proposed optimal outputs	-
$\mathbf{Y_{tr}}$	Output training matrix	-

References

1. Zakrzewski, J., Smalley, A.P., Kabeshov, M.A., Gaunt, M.J., and Lapkin, A.A. *Angew. Chem. Int. Ed.*, **2016**, *55*, 8878-83.
2. Zullo, L.C., Computer aided design of experiments. An engineering approach. PhD Thesis, Imperial College London, U.K., 1991.
